# Antibody Titers Against Human Cytomegalovirus gM/gN and gB Among Pregnant Women and Their Infants

**DOI:** 10.3389/fped.2022.846254

**Published:** 2022-06-23

**Authors:** Maria Talavera-Barber, Kaitlyn Flint, Brianna Graber, Ravi Dhital, Irina Kaptsan, Alexandra K. Medoro, Pablo J. Sánchez, Masako Shimamura

**Affiliations:** ^1^Avera McKennan Hospital and University Medical Center, Avera Research Institute, Sioux Falls, SD, United States; ^2^Center for Vaccines and Immunity, The Abigail Wexner Research Institute at Nationwide Children's Hospital, Columbus, OH, United States; ^3^Division of Neonatology, Department of Pediatrics, Nationwide Children's Hospital, The Ohio State University College of Medicine, Columbus, OH, United States; ^4^Division of Infectious Diseases, Department of Pediatrics, Nationwide Children's Hospital, The Ohio State University College of Medicine, Columbus, OH, United States; ^5^Center for Perinatal Research, The Abigail Wexner Research Institute at Nationwide Children's Hospital, Columbus, OH, United States

**Keywords:** congenital cytomegalovirus infection, prematurity, glycoprotein antibodies, seroimmunity, vaccines

## Abstract

Congenital CMV (cCMV) infection can affect infants born to mothers with preconceptional seroimmunity. To prevent cCMV due to nonprimary maternal infection, vaccines eliciting responses exceeding natural immunity may be required. Anti-gM/gN antibodies have neutralizing capacity *in-vitro* and in animal models, but anti-gM/gN antibodies have not been characterized among seroimmune pregnant women. Paired maternal and infant cord sera from 92 CMV seropositive mothers and their full-term or preterm infants were tested for anti-gM/gN antibody titers in comparison with anti-gB titers and neutralizing activity. Anti-gM/gN titers were significantly lower than anti-gB titers for all groups and did not correlate with serum neutralizing capacity. Further study is needed to determine if higher anti-gM/gN antibody titers might enhance serum neutralizing capacity among seropositive adults.

## Introduction

Congenital cytomegalovirus (cCMV) infection is the most common congenital viral infection, affecting approximately 0.5% of newborns in the United States (1, 2). Neurodevelopmental impairments and sensorineural hearing loss can affect children with cCMV infection, with substantial personal and societal consequences (1, 3–5). Preventing cCMV infection remains a major clinical challenge. Women with preconceptional seroimmunity have lower risk of vertically transmitting CMV compared to women lacking preconceptional seroimmunity, but due to high CMV seroprevalence among women of childbearing age worldwide, a greater total number of cCMV-infected infants are born to women with nonprimary infection (4, 6–9). For seropositive women, vaccines may need to elicit serological responses exceeding that of natural immunity to prevent cCMV transmission and/or damaging sequelae for fetuses infected *in utero*.

Current candidate vaccines target CMV glycoprotein B (gB), the pentameric complex (gH/gL/gpUL128-131), pp65, and the IE1/2 gene products (10). To prevent primary CMV infection among seronegative pregnant women, a CMV vaccine targeting gB was tested among CMV seronegative women of childbearing age in a Phase II clinical trial and showed a vaccine efficacy of 50% (11). This vaccine elicited virus-neutralizing anti-gB antibodies at lower titers than natural infection, and its efficacy was thought to be partially due to antibody-dependent cellular phagocytosis by non-neutralizing antibodies (12, 13). Antibodies against the pentameric complex have potent neutralizing activity *in-vitro* and in animal models and can be elicited by vaccination (14–17). However, anti-pentamer antibody titers did not differ between women who transmitted CMV congenitally to their infants and non-transmitting mothers (18).

In addition to gB and the pentameric complex, CMV also encodes a third surface glycoprotein complex consisting of glycoproteins M and N (gM/gN). Glycoprotein M is the most abundant component of the viral proteome and is complexed to glycoprotein N, which exhibits sequence variation and glycosylation consistent with immune evasion functions (19–22). This complex elicits antibodies with *in vitro* neutralizing capacity that are detectable among seropositive adults and in CMV hyperimmune globulin (19, 23–27). In mice, co-immunization with DNA vaccines encoding murine CMV (MCMV) gM and gN provided mice with complete protection against lethal MCMV challenge (28). These studies support the potential utility of including gM/gN antigens in an HCMV vaccine, but it is unknown whether such a vaccine could enhance humoral responses among seropositive individuals. Anti-gM/gN antibody titers during pregnancy and their transplacental transmission to the infant in mother-infant dyads delivered at various gestational ages have not been characterized. In this study, we utilized an institutional biorepository to quantify anti-gM/gN antibody titers in mother-infant dyads in comparison with anti-gB titers and neutralizing activity.

## Materials and Methods

### Patient Population

For this retrospective cohort study, subjects from the Ohio Perinatal Research Network Pediatric Research Repository (OPRN PRR) who had paired maternal and infant umbilical cord sera from singleton pregnancies were identified. Maternal third trimester sera were screened by enzyme-linked immunoassay (ELISA) for CMV IgM and IgG. For subjects with IgG+/IgM- third trimester results, the serostatus was confirmed using first trimester maternal sera to exclude women with primary infection during pregnancy. Seropositive mothers with sufficient maternal and infant sera available for analysis were included in this study. Informed consent was obtained for enrollment in the PRR through an IRB-approved protocol (NCH IRB10-00035). Demographic and clinical characteristics of mothers and infants were collected through the OPRN PRR.

### ELISA Assay

Expression plasmids encoding full-length HCMV ORF UL55 (gB), UL100 (gM) or UL73 (gN) (26) were transfected into 293T cells (American Type Culture Collection, Manassas VA) using Mirus LT-1 reagent (Mirus Bio LLC, Madison WI). Expression of gB or gM/gN was confirmed by immunofluorescence staining using monoclonal antibodies (mabs) for gB (IgG CH-28, Santa Cruz Biotechnology, Santa Cruz CA) or the gM/gN complex (IgM mab 14-16A, gift of W. Britt, University of Alabama at Birmingham). Transfected cell lysates were used for anti-gB or anti-gM/gN ELISAs and were validated using monoclonal antibodies and sera from known seropositive and seronegative individuals. The glycoprotein-specific antibody transplacental transmission ratio was calculated as the infant titer/maternal titer for each infant-mother dyad.

### Neutralization Assay

HCMV strain AD169 with a repaired mutation in UL128-131 and expressing green fluorescent protein (GFP) (gift of T. Shenk, Princeton University, Princeton NJ) was utilized for the neutralization assays. CMV was incubated with maternal or infant sera in 2-fold serial dilution, with and without guinea pig complement (CedarLane Laboratories Limited, Ontario Canada) prior to infecting fibroblasts in duplicate as previously described (26, 29, 30). For these assays, complement was included to identify potential differences in neutralizing capacity mediated by complement-fixing antibodies in comparison with non-complement-fixing antibodies (31) Control wells were infected with CMV, with or without complement. At 72 h post-infection, green fluorescent protein (GFP)-expressing cells were quantified using an EVOS cell imaging system (ThermoFisher) and Image J software (https://imagej.github.io/ website). Percent neutralization was calculated for each serum dilution relative to the positive control wells. The IC50 (inhibitory concentration 50) titer was defined as the titer at which ≥50% neutralization was observed.

### Statistical Analysis

Demographic characteristics were described as categorical or continuous variables (number, percent; median, range). The Mann-Whitney U test, Kruskal-Wallis test, or Wilcoxon signed rank test was used to compare two or more groups, accepting significance at *p* < 0.05. Linear regression was utilized to calculate the correlation coefficient between continuous variables. All analyses were conducted using Prism 9.0 (GraphPad, San Diego CA).

## Results

### Study Population Demographics

In this cohort of 92 mother-infant dyads (Table 1), 61 women (66%) delivered FT infants (range, 37–40 weeks), 24 (26%) delivered LPT infants (range, 34–36 weeks), and 7 (8%) delivered PT infants (range, 27–33 weeks). The mothers had a median age of 27 years (range, 18–38 years), 55 (60%) were African American, 6 (7%) were Hispanic, 56 (61%) were multiparous, and 71 (77%) had public insurance. There were no significant differences in maternal demographics by GA group. Among the infants, 46 (50%) were male, 67 (73%) were African American, and 6 (7%) were Hispanic.

**Table 1:** Maternal and infant demographics.

**Demographics**	**Preterm** **(≤33 weeks)**	**Late preterm** **(34–36 wks)**	**Full-term** **(≥37 weeks)**
Total per group, *n* (%)	7 (7.6)	24 (26)	61 (66)
**Maternal:**			
Age, years, median (range)	30 (18–35)	28 (20–38)	25 (18–36)
Race, *n* (%)			
Black	5 (71%)	17 (71%)	33 (54%)
Caucasian	-	2 (8%)	15 (46%)
Other	-	-	3 (5%)
Not reported	2 (29%)	5 (21%)	10 (16%)
Ethnicity, *n* (%)			
Non-hispanic	4 (57%)	21 (88%)	51 (84%)
Hispanic	-	2 (8%)	4 (7%)
Unknown	3 (43%)	1 (4%)	6 (10%)
Gravida > G1, *n* (%)	7 (100%)	22 (92%)	56 (92%)
Parity >P1, *n* (%)	6 (86%)	13 (54%)	37 (61%)
Public insurance, *n* (%)	5 (71%)	16 (67%)	50 (82%)
Education less than high school, *n* (%)	3 (43%)	2 (8%)	13 (21%)
**Infant:**			
Gender, male, *n* (%)	4 (57%)	10 (42%)	32 (52%)
Birth weight, grams, median (range)	1,301 (977–1,996)	2,492 (1,844–3,275)	3,216 (2,183–4,535)
Race			
Black	7 (100%)	20 (87%)	40 (66%)
Caucasian	-	1 (4%)	15 (24%)
Not reported	-	2 (8%)	5 (8%)
Other	-	1 (4%)	1 (2%)
Ethnicity			
Non-hispanic	7 (100%)	20 (87%)	58 (84%)
Hispanic	0	2 (9%)	4 (6%)

### Anti-gB Titers Are Higher Than Anti-gM/gN Titers

**Figure 1:**
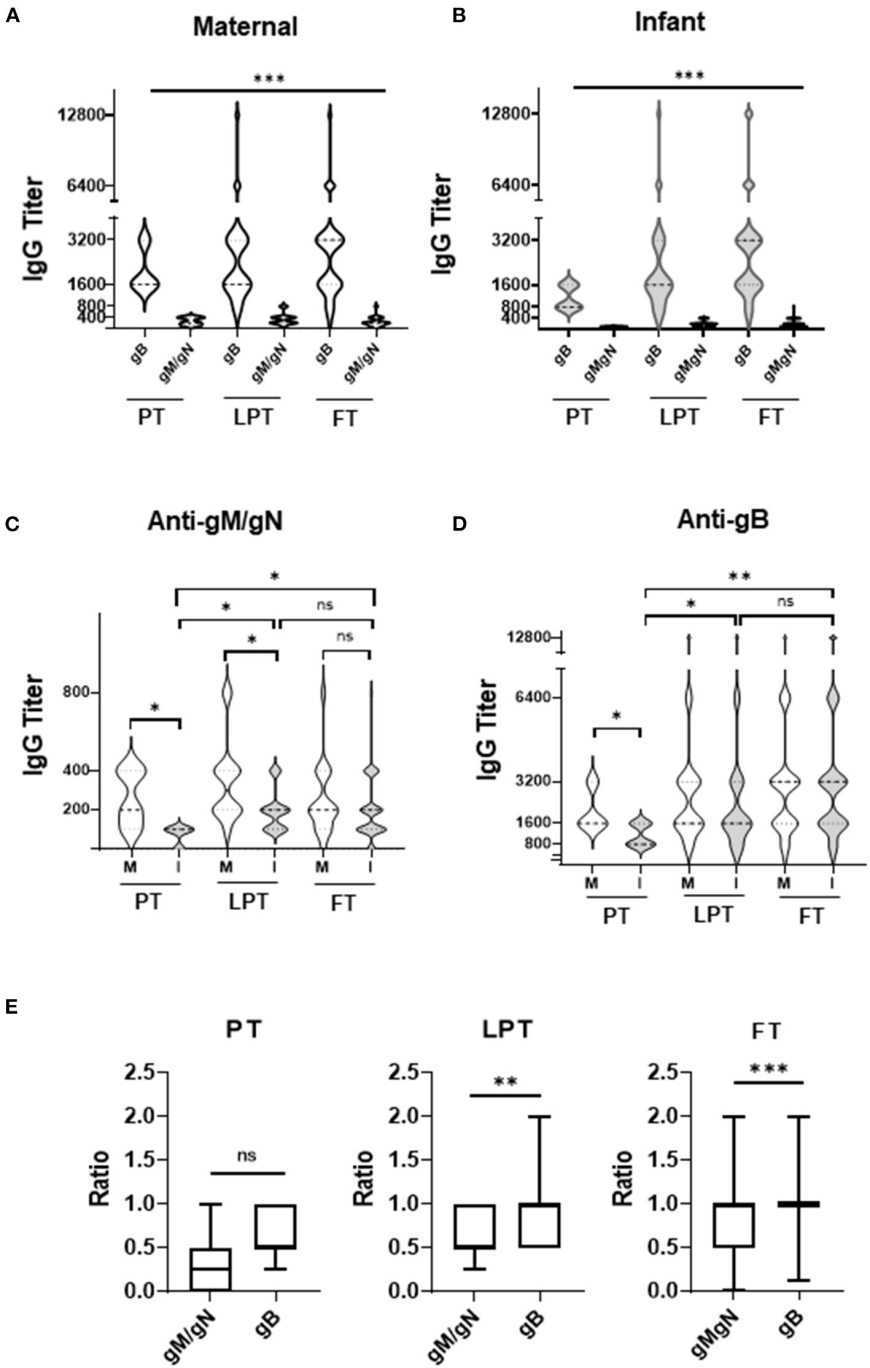
Anti-gM/gN and anti-gB antibody titers among mother-infant dyads. **(A,B)** CMV anti-gM/gN antibodies and anti-gB antibodies were quantitated by ELISA for mothers **(A)**, and infants **(B)**. **(C,D)** Antibody titers against gM/gN **(C)** or gB **(D)** were compared for preterm (PT), late-preterm (LPT), and full-term (FT) mother (M) and infant (I) groups. **(E)** Transplacental transmission ratio between maternal 3^rd^ trimester and infant cord blood of anti-gB and anti-gM/gN antibody titers were calculated for all gestational ages (PT, LPT, FT). Data is shown as the median value with min-max ranges plotted. ns, not significant; ^*^p < 0.05; ^**^p < 0.01; ^***^p < 0.001.

For all mothers and infants, anti-gB titers were significantly higher than anti-gM/gN titers (Figures 1A,B, *p* < 0.001) at all gestational ages (PT, LPT, FT). Anti-gM/gN titers were significantly lower among PT and LPT infants compared to their mothers, but were similar for FT infants and mothers (Figure 1C). Anti-gB titers were significantly lower among PT infants compared to their mothers, but were similar between mother-infant dyads for the LPT and FT groups (Figure 1D). Comparing anti-gM/gN titers among infant groups (Figure 1C), PT infants had significantly lower titers than LPT and FT infants, but the latter two groups were similar. Similar results were observed for anti-gB titers (Figure 1D), with those of PT infants significantly lower than LPT and FT groups. Antibody titer ranges and statistical comparisons are summarized in Table 2. To determine the efficiency of transplacental transmission by gestational age, a transmission ratio was calculated for anti-gM/gN and anti-gB antibodies (Figure 1E).

**Table 2:** Antibody titers among preterm, late-preterm and full term mothers and infants.

	**Anti-gM/gN titers**	**Anti-gB titers**	*p*-value *
**Maternal**			
Preterm (PT)	200 [100–400]	1,600 [1,600–3,200]	*p* = 0.0006
Late-preterm (LPT)	300 [100–800]	1,600 [800–12,800]	*p* < 0.0001
Full-term (FT)	200 [100–800]	3,200 [200–12,800]	*p* < 0.0001
***p*****-value** **	*p* = 0.0496	*p* = 0.6044 (n.s.)	
**Infant**			
Preterm (PT)	100 [0–100]	800 [800–1,600]	*p* = 0.0006
Late-preterm (LPT)	200 [100–400]	1,600 [400–12,800]	*p* < 0.0001
Full-term (FT)	200 [100–800]	3,200 [400–12,800]	*p* < 0.0001
***p*****-value** **	*p* = 0.0145	*p* = 0.0046	

*All titers shown as median [min-max].*

*n.s., not significant.*

* *Mann-Whitney U test.*

** *Kruskal-Wallis test*.

PT infants had similar transplacental transmission ratios for anti-gM/gN and anti-gB antibodies, whereas LPT and FT infants had significantly lower ratio for anti-gM/gN antibodies compared to anti-gB antibodies. These results indicate that anti-gM/gN antibody titers are significantly lower than anti-gB titers among both mothers and infants independently of gestational age, but glycoprotein-specific antibodies are lower among PT infants than LPT and FT infants. Transplacental transmission of anti-gM/gN antibodies is lower than anti-gB antibodies among LPT and FT infants, possible related to the overall higher maternal anti-gB titers.

### Neutralization Assays

**Figure 2:**
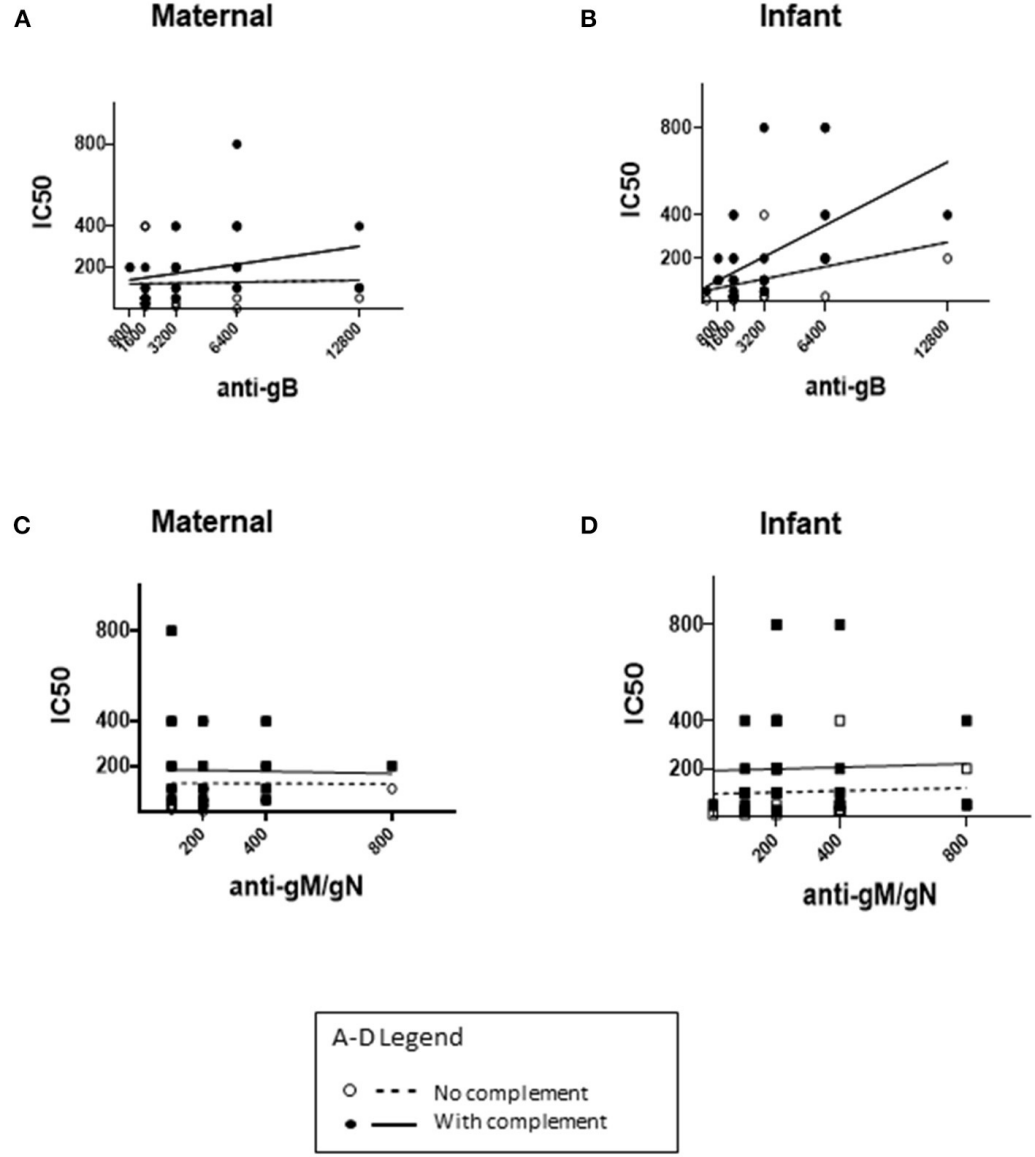
Neutralizing titers of maternal and infant sera. CMV neutralizing capacity of maternal third trimester sera and infant cord sera were tested using an *in-vitro* neutralizing assay, without or with guinea pig complement, and the IC50 titer was calculated for each sample. The IC50 titers without complement (dotted lines) and with complement (solid lines) were compared to anti-gB and anti-gM/gN titers in maternal **(A,C)** and infant **(B,D)** sera. **(A,B)** The x-axis range reflects anti-gB titers ranging from 1:200-1:12800. **(C,D)** The x-axis range reflects anti-gM/gN titers ranging from 0–1:800 **(A–D)**. For each correlation plot, a single circle/square may represent more than one sample with the antibody titer/neutralizing IC50 titer shown in each graph.

The neutralizing capacity of maternal and infant sera was tested in serial dilution, with and without complement (+/- complement), and neutralizing titers compared for mother-infant dyads (Figure 2). Complement was included to identify any enhancement of neutralizing capacity conferred by complement-fixing antibodies as compared with complement non-fixing antibodies, which has been described for both anti-gB and anti-gM/gN antibodies (26, 29, 32). Comparison of neutralizing titers with anti-gB or anti-gM/gN titers among mothers (Figures 2A,C) or infants (Figures 2B,D) showed modest correlation of complement-fixing neutralizing titers with anti-gB titers for mothers (R = 0.0163) and infants (R = 0.0453), but no correlation with anti-gM/gN titers. Together, these data show that anti-gM/gN titers were low for mothers and infants, and that serum neutralizing capacity did not correlate with anti-gM/gN titers.

## Discussion

Congenital CMV (cCMV) infection can occur during nonprimary infection among mothers with preconceptional seroimmunity. A maternal vaccine against gB has been evaluated among seronegative women of childbearing age, but it has not been tested among the larger population of seropositive women who are also at risk to transmit CMV during nonprimary infection or viral reactivation. Antibodies against gB are present at high quantities among seropositive individuals and are associated with serum neutralizing capacity, suggesting that increasing anti-gB antibodies by vaccination may provide only incremental improvement in protection among seropositive women (33, 34). Our results confirm that anti-gB titers correlate to some extent with serum neutralizing capacity among mothers and their infants.

In contrast, anti-gM/gN antibody titers were lower than anti-gB titers among pregnant women and their infants independent of gestational age and did not correlate with serum neutralizing capacity. Transplacental transmission of anti-gM/gN antibodies to late preterm and full term infants was significantly lower than that of anti-gB antibodies. Human anti-gM/gN antibodies have been shown to neutralize CMV infection *in-vitro* and in a murine model but, similar to our findings, a recent study of non-pregnant CMV seropositive adults showed that anti-gM/gN titers did not correlate with *in-vitro* serum neutralizing activity (25, 26, 28). The reasons for the discrepancy between *in-vitro* and animal model data compared to clinical serologic responses are unclear, and it is unknown whether increasing human anti-gM/gN titers could improve serum CMV-neutralizing potential. As anti-gM/gN antibodies can be elicited in animal models after DNA immunization, it remains a possibility that vaccination could be used to enhance anti-gM/gN titers among human populations, including those with natural seroimmunity to HCMV infection, although the resulting impact upon serum neutralizing capacity is not yet defined.

A limitation of this study is that only one virus strain, AD169, was utilized for all assays. AD169 encodes gN genotype 1, so gN antibodies specific for genotypes 2–4 may not be detected in our ELISA assay. However, 70% of seropositive adults have anti-gN antibodies that react with epitopes present in all gN genotypes, whereas 30% of adults have genotype-specific anti-gN antibodies (20, 35). Our assay would detect the 70% of individuals with anti-gN antibodies common to all genotypes, as well as those with gN-1 specific antibodies, thus capturing the majority of anti-gN serologic responses in our cohort. Future studies could better define individual differences in gN genotype-specific titers in order to determine the potential utility of including all 4 gN genotypes in a gM/gN vaccine.

As this cohort was derived from a biorepository, the patients were not selected to represent the demographic of the general U.S. population. Previous research has demonstrated socioeconomic and racial/ethnic disparities in CMV seropositivity with 81.7% for Hispanics, 75.8% for African Americans, and 51.2% for Non-Hispanic whites in the United States (36–38). Similarly, cCMV rates are higher for nonwhites than whites and individuals with lower socioeconomic (SES) backgrounds compared with those of higher SES (39, 40). Our cohort of seropositive women were predominantly African American and non-Hispanic, with 77% on public insurance. These results align with the known SES and racial disparity in CMV seroprevalence in the US. However, the demographics of this cohort differs from the majority of the U.S. population, which could confer an unknown bias in our results and limit the generalizability of our findings to other demographic populations.

Finally, in this cohort, anti-CMV IgM was not detected among any of the cord blood samples (data not shown), indicating that no infants had congenital CMV infection. Congenital CMV transmission is reported among 0.5% of U.S. populations (41), so we would expect transmission to <1 infant (0.46) among 92 mothers. It is therefore not surprising that congenital CMV was not present in this cohort. However, consequently, no conclusions can be drawn regarding the protective benefit of anti-gB and ant-gM/gN antibodies against congenital CMV transmission among this study cohort.

In summary, among CMV seropositive pregnant women and their newborn infants, anti-gM/gN titers are lower than anti-gB titers and do not correlate with serum neutralizing capacity. Further study is needed to determine if higher human anti-gM/gN antibody titers may confer improved serum neutralizing capacity against CMV infection.

## Data Availability Statement

The raw data supporting the conclusions of this article will be made available by the authors, without undue reservation.

## Ethics Statement

The studies involving human participants were reviewed and approved by IRB-approved protocol (NCH IRB10-00035). Written informed consent to participate in this study was provided by the participants' legal guardian/next of kin.

## Author Contributions

MT-B and MS designed the study. MT-B, KF, BG, RD, IK, and MS collected, processed, and analyzed the data. MT-B, AM, PS, and MS had major contributions to writing the manuscript. All authors participated in the revision of the manuscript.

## Funding

This study was funded by the National CMV Foundation (MTB) and the Abigail Wexner Research Institute at Nationwide Children's Hospital (MS and PS). The Ohio Pediatric Research Network Pediatric Research Repository (OPRN PRR) was funded by the Abigail Wexner Research Institute at Nationwide Children's Hospital.

## Conflict of Interest

The authors declare that the research was conducted in the absence of any commercial or financial relationships that could be construed as a potential conflict of interest.

## Publisher's Note

All claims expressed in this article are solely those of the authors and do not necessarily represent those of their affiliated organizations, or those of the publisher, the editors and the reviewers. Any product that may be evaluated in this article, or claim that may be made by its manufacturer, is not guaranteed or endorsed by the publisher.
